# Regulation of Lean Mass, Bone Mass, and Exercise Tolerance by the Central Melanocortin System

**DOI:** 10.1371/journal.pone.0042183

**Published:** 2012-07-27

**Authors:** Theodore P. Braun, Benjamin Orwoll, Xinxia Zhu, Peter R. Levasseur, Marek Szumowski, My Linh T. Nguyen, Mary L. Bouxsein, Robert F. Klein, Daniel L. Marks

**Affiliations:** 1 Papé Family Pediatric Research Institute, Oregon Health & Science University, Portland, Oregon, United States of America; 2 MD/PhD Program, Oregon Health & Science University, Portland, Oregon, United States of America; 3 Bone and Mineral Unit, Division of Endocrinology, Diabetes and Clinical Nutrition, Oregon Health & Science University, Portland, Oregon, United States of America; 4 Department of Pediatrics, Loma Linda University, Loma Linda, California, United States of America; 5 Center for Advanced Orthopedic Studies, Beth Israel Deaconess Medical Center and Harvard Medical School, Boston, Massachusetts, United States of America; University of Sao Paulo, Brazil

## Abstract

Signaling via the type 4-melanocortin receptor (MC4R) is an important determinant of body weight in mice and humans, where loss of function mutations lead to significant obesity. Humans with mutations in the MC4R experience an increase in lean mass. However, the simultaneous accrual of fat mass in such individuals may contribute to this effect via mechanical loading. We therefore examined the relationship of fat mass and lean mass in mice lacking the type-4 melanocortin receptor (MC4RKO). We demonstrate that MC4RKO mice display increased lean body mass. Further, this is not dependent on changes in adipose mass, as MC4RKO mice possess more lean body mass than diet-induced obese (DIO) wild type mice with equivalent fat mass. To examine potential sources of the increased lean mass in MC4RKO mice, bone mass and strength were examined in MC4RKO mice. Both parameters increase with age in MC4RKO mice, which likely contributes to increases in lean body mass. We functionally characterized the increased lean mass in MC4RKO mice by examining their capacity for treadmill running. MC4R deficiency results in a decrease in exercise performance. No changes in the ratio of oxidative to glycolytic fibers were seen, however MC4RKO mice demonstrate a significantly reduced heart rate, which may underlie their impaired exercise performance. The reduced exercise capacity we report in the MC4RKO mouse has potential clinical ramifications, as efforts to control body weight in humans with melanocortin deficiency may be ineffective due to poor tolerance for physical activity.

## Introduction

The type 4-melanocortin receptor (MC4R) is a known regulator of somatic growth. Genetic blockade [1] or loss of function of the MC4R results in increased linear growth in rodents [2] and humans [3]. Interestingly, humans with MC4R deficiency have a lower body fat content, and therefore higher lean mass when compared to leptin-deficient individuals with similar body mass index (BMI). MC4RKO mice resist lean mass loss due to tumor growth [4] and chronic renal failure [5]. Furthermore, pharmacologic blockade of the MC4R attenuates lean mass loss in a multitude of catabolic conditions [4]–[8]. Despite this association, a thorough characterization of the relationship between melanocortin signaling and lean mass has not been performed.

Increased bone mineral density has also been reported in humans [3] and animals [9] with MC4R deficiency, suggesting that increases in bone mass may account for some of the lean mass phenotype seen in these situations. Complicating the interpretation of increased lean mass in melanocortin deficiency is the compensatory muscle hypertrophy and bone mineralization that occur in parallel with increasing fat mass. As body weight increases, muscle and bone mass increase, presumably in response to increased mechanical loading. Therefore, we examined the body composition of MC4RKO and diet-induced obese (DIO) mice to investigate the role of melanocortin signaling in regulating the accrual of lean mass independent from increases in fat mass.

Our results show that young MC4RKO mice initially have an increase in fat mass without changes in lean mass relative to WT mice. However, increased lean mass rapidly develops relative to DIO-WT mice, which display an equivalent rate of fat mass accrual. This increased lean mass is associated with a decreased exercise performance independent of increased body weight and with increased grip strength. Additionally, MC4RKO mice have a late onset increase in bone mass and strength. Collectively, these results demonstrate that melanocortin signaling is a fundamental determinant of lean body mass, a component of which includes bone mass.

## Results

### MC4R Deficiency Increases the Accrual of Lean Mass Independent from Changes in Fat Mass

**Figure 1:**
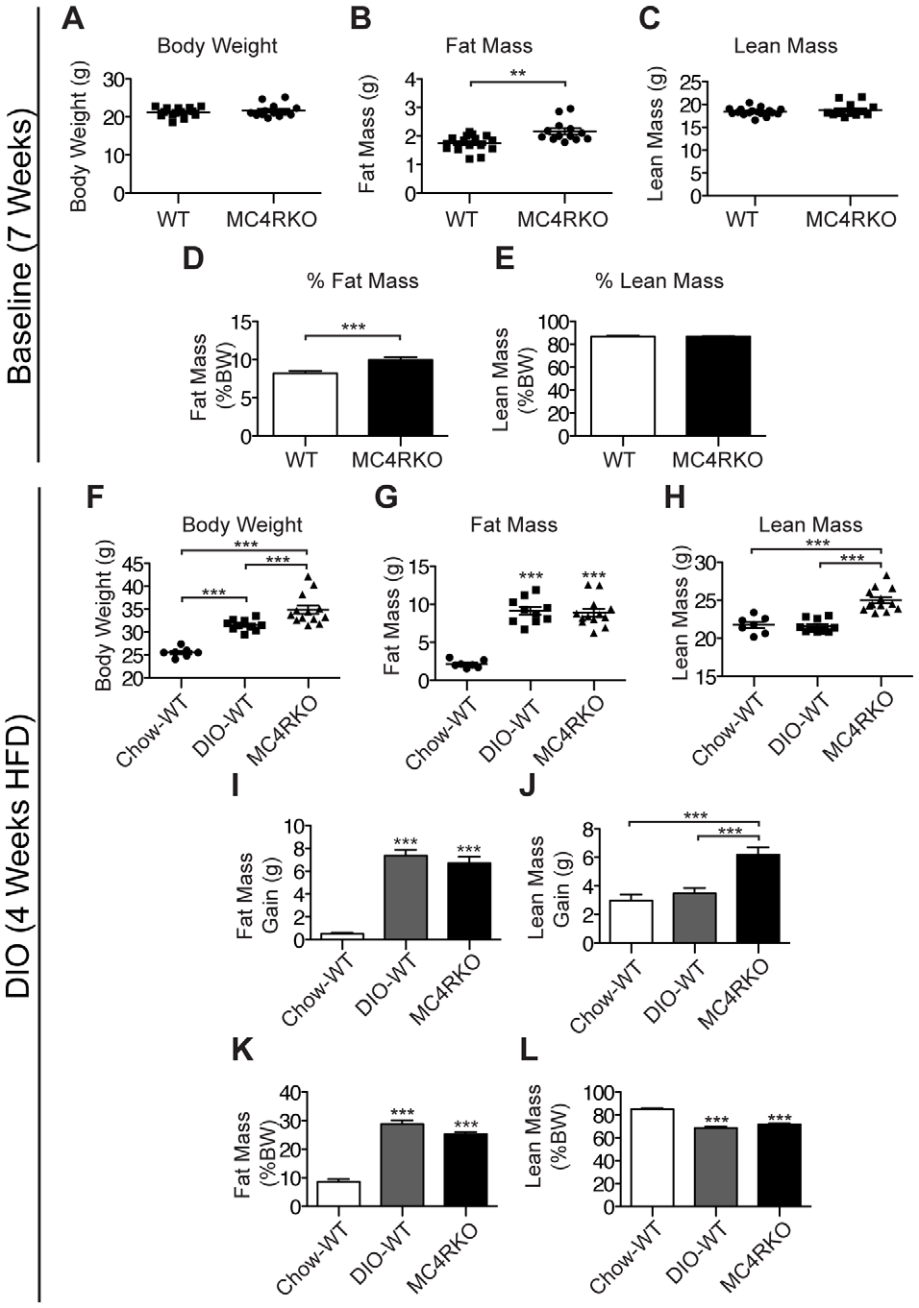
MC4RKO Mice Rapidly Accrue Lean Mass Independent from Fat Mass. (**A**) Body weights of 7-week-old male MC4RKO mice (n = 20) were compared with age and weight matched WT mice (n = 10). (**B**) MC4RKO mice have increased fat mass, (**C**) but normal lean mass. (**D**) Fat mass as a percentage of body weight (BW) is increased in MC4RKO mice. (**E**) Lean mass as a percentage of body weight is unaltered in MC4RKO mice. A subset of WT mice (n = 13) were placed on HFD (DIO-WT) for 4 weeks, from 8–12 weeks of age, while the remainder (n = 7) were maintained on a low fat control diet (Chow-WT). MC4RKO mice were maintained on a low fat control diet during this time. (**F**) MC4RKO mice weighed more at 12 weeks of age than DIO-WT mice. (**G)** MC4RKO mice have equivalent fat mass at 12 weeks of age compared with DIO-WT mice. (**H**) MC4RKO mice have increased lean mass relative to DIO-WT mice at 12 weeks of age. (**I**) Mice fed a HFD for 4 weeks gain an equivalent amount of fat mass as MC4RKO mice. (**J**) MC4RKO mice gain more lean mass than DIO-WT mice. (**K**) % body fat, (**L**) and % lean mass. Data represented as mean ± s.e.m. ** = P<0.01, *** = P<0.001 as calculated by unpaired t-test or one-way ANOVA with a Bonferroni post test.

To examine the time course of the increased lean mass phenotype in MC4RKO mice, we examined young WT and KO mice at 7 weeks of age. At this time point, MC4RKO mice were not yet significantly heavier than WT mice (21.7±0.5 g for KO vs. 21.2±0.3 g for WT, p = 0.09) (Figure 1 A). Interestingly, fat mass was increased in MC4RKO relative to WT mice (2.2±0.1 g for KO vs. 1.7±0.1 g for WT), with no differences evident in lean mass (18.8±0.4 g for KO vs. 18.4±0.2 g for WT) (Figure 1 B, C). This resulted in an increased fat mass percentage and a decreased lean body mass percentage in KO mice relative to WT mice (Figure 1 D, E).

We next explored whether an increased lean mass phenotype would occur with aging in the MC4RKO mice. To control for the effects of increasing body weight and fat mass, we placed a subset of WT control mice on a high fat diet (HFD) for 4 weeks to generate DIO, while the remainder of the WT animals were maintained on low-fat chow (Chow-WT). Despite DIO-WT mice gaining significant weight over the month of high fat feeding relative to Chow-WT mice, chow-fed MC4RKO mice gained more weight and were significantly heavier at 12 weeks of age (34.9±0.9 g for KO vs. 31.5±0.4 g for DIO-WT vs. 25.6±0.4 for Chow-WT) (Figure 1 F). HFD produced a significant increase in fat mass in WT mice over the 4-week period relative to Chow-WT mice. This increase paralleled the fat mass gain in chow-fed MC4RKO mice (6.7±0.5 g for KO vs. 7.4±0.5 g for DIO-WT vs. 0.5±0.1 g for Chow-WT) (Figure 1 G, I). As a result, at the end of the 4-week period, WT-DIO and MC4RKO mice exhibited equivalent fat mass (8.9±0.5 g for KO vs. 9.1±0.5 g for WT vs. 2.2±0.2 g for Chow-WT). Both WT-Chow and WT-DIO mice gained equivalent fat mass over the course of 4 weeks, however, MC4RKO mice gained nearly double the lean mass over the one-month period (6.2±0.5 g for KO vs. 3.5±0.4 g for WT vs. 3.0±0.4 for Chow-WT) (Figure 1 H, J). This resulted in a significantly increased lean mass (25.0±0.4 g for KO vs. 21.6±0.3 g for WT vs. 21.8±0.4 g for Chow-WT) in MC4RKO mice relative to DIO-WT mice (Figure 1 H, K, L). When controlling for body weight by only considering the 5 heaviest DIO-WT mice and the 5 lightest MC4RKO mice the differences in body composition become even more pronounced, with MC4RKO mice displaying 25% less fat mass than weight-matched DIO-WT mice.

### MC4RKO Mice Exhibit Increased Grip Strength and Decreased Exercise Performance

**Figure 2:**
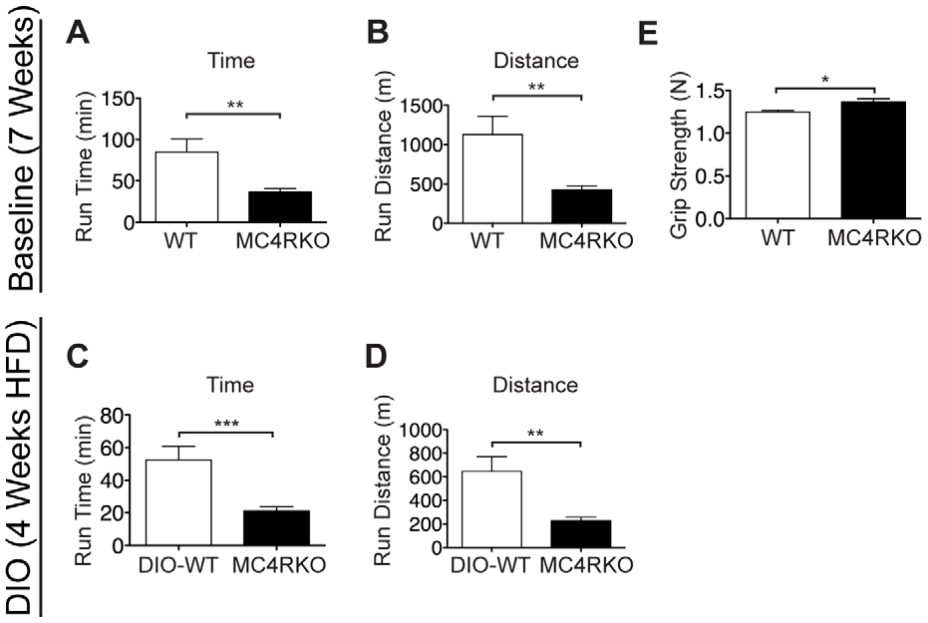
The type 4-melanocortin receptor is a critical regulator of exercise performance. 8-week-old male MC4RKO mice (n = 13) and age and weight matched WT controls (n = 10) were subjected to treadmill running at escalating speed. (**A**) MC4RKO mice showed decreased total treadmill run time. (**B**) MC4RKO mice showed decreased treadmill run distance. No correlation between run time or run distance with body weight was observed. WT mice (n = 13) were placed on HFD (DIO-WT) for 4 weeks until 12 weeks of age. MC4RKO mice were maintained on a low fat control diet. After this time, treadmill endurance was assessed. (**C**) MC4RKO showed a significantly attenuated run time (**D**) and run distance compared with WT mice. There was no significant correlation between run time or run distance and body weight. (**E**) Grip strength was also measured prior to the initiation of high fat feeding, and was increased in MC4RKO mice. Data represented as mean ± s.e.m. * = P<0.05, ** = P<0.01, *** = P<0.001 as calculated by unpaired t-test.

To examine the endurance capacity of MC4RKO mice, they were subjected to treadmill running. Despite only mild differences in body composition, MC4RKO mice showed a dramatic reduction in treadmill running time, and distance covered (Figure 2 A, B). MC4RKO mice ran for less than half the time and covered only one third of the distance achieved by WT mice. There was no significant correlation between body weight and either run time or distance covered in WT or MC4RKO mice (p = 0.2 for WT and p = 0.3 for MC4RKO). The endurance performance of MC4RKO mice was examined again relative to DIO-WT mice at 12 weeks of age, and again a significantly reduced run time and total distance traveled was observed in MC4RKO mice (Figure 2 C, D). BW was not a determining factor in the running performance of MC4RKO mice as it showed no significant correlation with run time or total distance covered for either WT or MC4RKO mice (p = 0.8 for WT, p = 0.8 for MC4RKO). To determine if a functional deficit in skeletal muscle might be responsible for the poor exercise endurance phenotype of MC4RKO mice, we measured grip strength and found that it was slightly increased in MC4RKO mice relative to WT mice (Figure 2 E). These data suggest that melanocortin signaling plays a fundamental role in determining skeletal muscle function.

### MC4RKO Mice Have Normal Muscle Fiber Composition and a Low Resting Heart Rate

**Figure 3:**
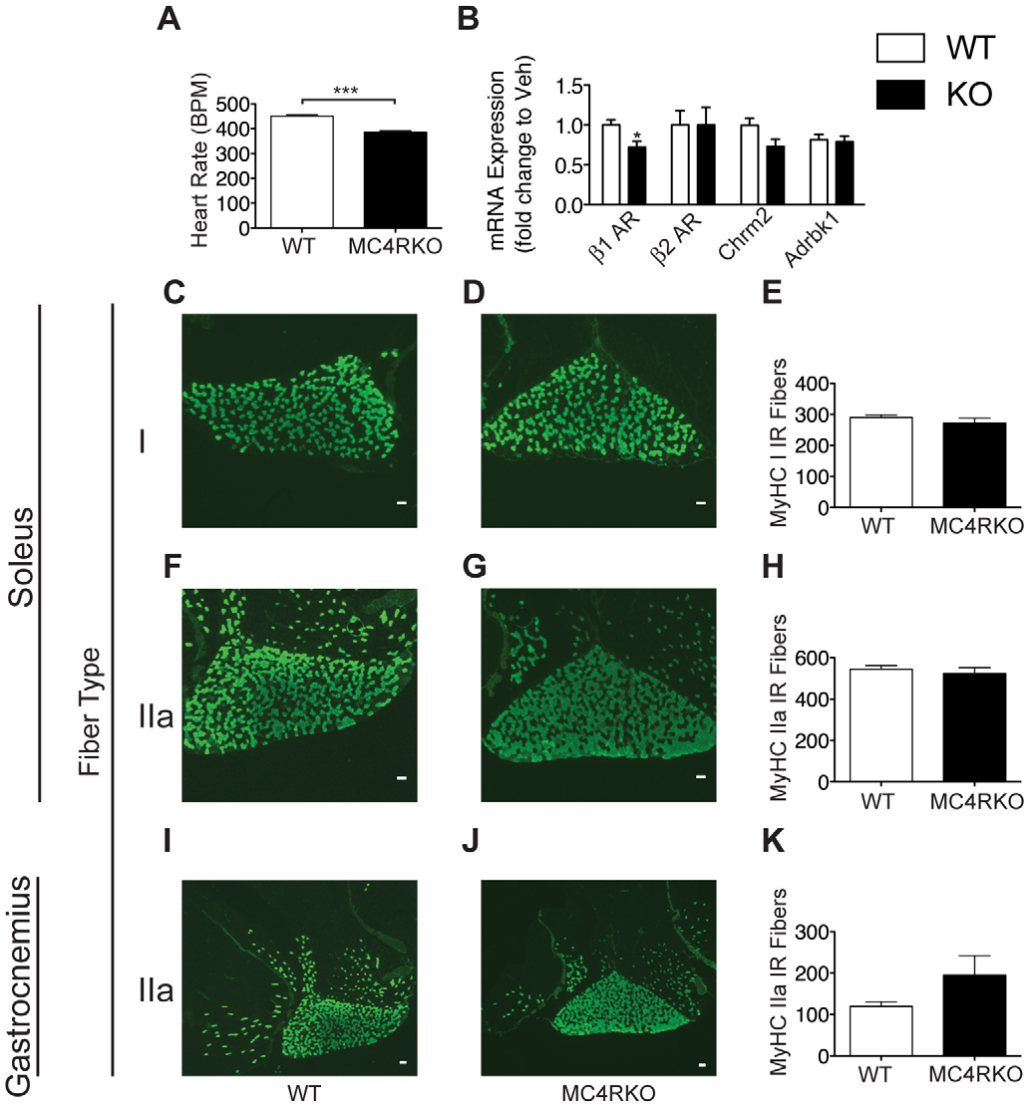
Mice lacking the type 4 melanocortin receptor have a normal muscle fiber type distribution with a decreased resting heart rate. Skeletal and cardiac muscle was examined in in 11-week old male WT (n = 5) and MC4RKO (n = 6) mice. (**A**) Resting heart rate was decreased in MC4RKO mice. (**B**) Cardiac gene expression in male MC4RKO and aged matched control mice at 35 weeks of age (n = 5–6/group). (**C–E**) No differences were seen in type I fiber number in the soleus, (**F–H**) type IIa fibers in the soleus or (**I–K**) type IIa fibers in the gastrocnemius. Scale bar = 100 µm. * = P<0.05, *** = P<0.001 as calculated by unpaired t-test.

To examine the possible causes for the reduced running performance in the MC4RKO mouse, we examined heart rate in MC4RKO mice. We found a significant reduction in heart rate in MC4RKO mice relative to WT controls, suggesting that impaired cardiac performance underlies the endurance deficit in these animals (Figure 3 A). To further explore this finding, we examined the expression of β1 and β2 adrenergic receptors in cardiac muscle (Figure 3 B). While the expression of the β2 adrenergic receptor was unchanged in MC4RKO mice, the β1 receptor was decreased in expression. We additionally examined the expression of β1 adrenergic receptor kinase (Adrbk1), which regulates β1 receptor signaling. No differences in Adrbk1 expression were observed between genotypes. Finally, expression of the type 2 muscarinic cholinergic receptor (Chrm2) was also examined, which underlies parasympathetic suppression of heart rate. While a trend toward decreased expression was seen in MC4RKO mice, this did not reach the level of statistical significance (p = 0.052). The decreased expression of the β1 adrenergic receptor is of particular interest, as decreased signaling via this receptor could potentially explain the decreased heart rate seen in MC4RKO mice. We also examined the fiber composition of the gastrocnemius and soleus muscles, as alterations in the relative abundance of glycolytic versus oxidative fibers could potentially explain the poor exercise endurance phenotype in the MC4RKO mouse. The rodent gastrocnemius is composed of predominantly type IIb glycolytic fibers, with scattered centrally located type IIa intermediate fibers [10]. In contrast the mouse soleus is made up of predominantly type IIa fibers at approximately a 2∶1 ratio with type I oxidative fibers. We saw no differences in the numbers of type IIa immunoreactive fibers in the gastrocnemius or soleus, and also saw no differences in the number of type I immunoreactive fibers in the soleus between genotypes (Figure 3 B–J). This suggests that alterations in fiber composition are not responsible for the reduced exercise performance in MC4RKO mice.

### Aged MC4RKO mice have increased bone mass and strength

Bone mass is known to be elevated in MC4RKO mice and skeletal mass is a major contributor to whole body lean mass. Therefore, we examined the skeletal phenotype in middle aged (20 weeks) and old (40 weeks) MC4RKO mice. Despite the significant discrepancy in overall body size between the MC4RKO and WT mice, there were no observed differences in femoral length at either 20 or 40 weeks of age (Tables 1 and 2). In 20-week-old MC4RKO mice, whole body and femoral bone mineral density (BMD) were significantly increased (by 6% and 11%, respectively) as compared to WT mice. By 10 months of age the markedly increased body size of the MC4RKO mice precluded assessment by the pixiMus® densitometer, but femoral and L5 vertebral BMD were significantly increased (by 11% and 13%, respectively) as compared to WT mice.

**Table 1:** Skeletal Phenotype of WT and MC4RKO Mice at 20 Weeks.

Age = 20 Weeks		WT	MC4RKO	*p* value
**Whole Body**	Weight (g)	22.2±0.3	31.5±1.5	<0.0001
	Length (cm)	9.51±0.10	9.68±0.09	NS
	BMD (mg/cm^2^)	49.2±0.4	52.0±0.8	<0.05
**Femur**	Length (mm)	15.5±0.01	15.9±0.01	NS
	BMD (mg/cm^2^)	48.7±0.5	54.1±1.0	<0.0005
	Ct. Ar. (mm^2^)	0.68±0.01	0.72±0.03	NS
	Ct. Th. (mm)	0.16±0.003	0.17±0.006	NS
	Ixx (mm^4^)	0.10±0.004	0.11±0.007	NS
	Ult. Force (N)	15.4±0.6	17.6±1.2	NS
	Stiffness (N/mm)	100.3±2.4	105.9±5.0	NS

The skeletal phenotype of twenty-week-old female WT (n = 14) and MC4RKO mice (n = 9) was examined by DEXA and microtomography. Bone strength was determined by 3 point bending to failure. Values are represented as mean ± SEM. Statistical significance was assessed by Students t-test, with significant differences assigned a p value<0.05.

**Table 2:** Skeletal Phenotype of WT and MC4RKO Mice at 40 Weeks.

Age = 40 weeks		WT	MC4RKO	*p* value
**Whole Body**	Weight (g)	24.5±0.4	61.4±2.1	<0.0001
	Length (cm)	9.44±0.07	10.3±0.05	<0.0001
**Femur**	Length (mm)	16.1±0.05	16.2±0.05	NS
	BMD (mg/cm^2^)	51.7±0.8	57.6±0.6	<0.0001
	Ct. Ar. (mm^2^)	0.76±0.02	0.91±0.02	<0.0001
	Ct. Th. (mm)	0.18±0.004	0.20±0.004	<0.0001
	Ixx (mm^4^)	0.12±0.004	0.18±0.006	<0.0001
	Ult. Force (N)	19.5±0.6	25.0±0.8	<0.0001
	Stiffness (N/mm)	117.4±4.5	142.5±4.3	<0.0001
**L5 Vertebra**	BMD (mg/cm^2^)	43.1±0.6	48.8±0.7	<0.0001
	BV/TV (%)	20.7±0.8	25.7±1.0	<0.005
	Tb No	3.4±0.1	4.4±0.1	<0.0001
	Tb Th	60.8±1.0	61.5±0.7	N.S.
	Conn Dens	86±7	135±7	<0.0001

The skeletal phenotype of forty-week-old female WT (n = 11) and MC4RKO mice (n = 12) was examined by DEXA and microtomography. Bone strength was determined by 3 point bending to failure. Vertebral morphometric variables describing bone microstructure were computed using direct 3D methods. Values are represented as mean ± SEM. Statistical significance was assessed by Students t-test, with significant differences assigned a p value<0.05.

**Figure 4:**
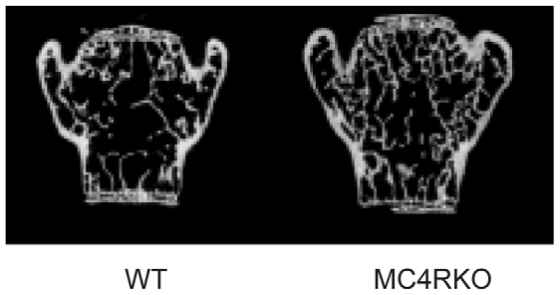
Trabecular bone microarchitecture of WT and MC4RKO mice at 40 Weeks. Two-dimensional frontal planar *μ*CT images of the fifth vertebral body were obtained as described in the Methods sections.

Although trends existed in bone strength and geometry, there were no statistical differences in femoral geometry or strength at 20 weeks of age (Table 1). However, by 40 weeks of age MC4RKO mice exhibited 19% greater femoral cortical area, 13% greater cortical thickness and 45% greater moment of inertia than WT mice (Table 2). These differences in femoral geometry were accompanied by a 29% increase in ultimate failure load and 21% increase in stiffness (Table 2). In addition to the femoral morphological differences, the vertebral BV/TV and trabecular number of 10-month-old MC4RKO L_5_ vertebrae compared to WT were 13% and 28% greater, respectively. Consistent with the increased vertebral bone volume and trabecular number, MC4RKO mice exhibited a 57% increase in trabecular connectivity density compared to WT mice. Representative two-dimensional frontal planar *μ*CT images illustrating the differences in bone integrity of the fifth lumbar vertebral body between WT and MC4RKO mice are presented in Figure 4. These data demonstrate that MC4RKO mice have increased bone mass that evolves in concert with the development of severe obesity.

## Discussion

Increased lean mass has been associated with MC4R mutations in humans [3], [11] and melanocortin blockade protects against lean mass loss in experimental cachexia in rodents [6], [12], [13]. Furthermore, MC4RKO mice exhibit increased body length, suggesting a role for signaling at the MC4R in restraining somatic growth [2]. Obesity results in an increase in both fat and lean body mass, although fat mass increases to a larger extent, increasing the fat: lean ratio. While MC4RKO loss of function has been associated with increased lean mass for a given BMI, a thorough characterization of body composition has yet to be performed to allow for the deconvolution of increasing adiposity from alterations in lean mass [4], [7], [14], [15]. Furthermore, while it has been suggested that an increase in lean mass associated with MC4R blockade is the result of increased muscle mass, the function of skeletal muscle in this setting has not been examined. This work demonstrates that MC4RKO mice accumulate lean mass at an accelerated rate independent from the accumulation of fat mass. While MC4R deficiency in humans is associated with increased lean mass relative to leptin deficient individuals, we confirm the association between MC4R loss-of-function and increased lean mass in MC4RKO mice relative to DIO mice.

Multiple lines of evidence suggest that signaling via the MC4R can influence metabolic pathways in peripheral tissues via the autonomic nervous system [16], [17]. The regulation of adipose tissue by melanocortin signaling has been extensively characterized, where signaling at the MC4R stimulates lipolysis. However, the contribution of melanocortin signaling to the regulation of lean tissue mass has not been described. In addition to the potential regulation of lean mass by the autonomic nervous system, melanocortin signaling regulates the levels of numerous hormones. Both insulin and circulating IGF-1 levels are elevated in humans with MC4R mutations, which would be expected to exert powerful trophic effects on muscle [18]. In total, these features of MC4R deficiency would be expected to promote the accrual of lean mass.

Bone mass represents another significant component of total lean mass. Elevated bone mass was found in multiple cohorts of MC4R deficient humans, and in MC4RKO mice [9]. Furthermore, MC4R deficient humans display isolated decreases in circulating markers of bone resorption, without alterations in markers of bone formation [9]. Consistent with these findings, we found significant elevations in bone mineral density in 5-month and 10-month-old MC4RKO mice. At both time points examined, significant elevations in body weight were already present in MC4RKO mice. Some controversy exists as to whether obesity is associated with increased bone mass [19]. Obese individuals demonstrate increased bone mass. However, when the effects of mechanical loading due to increased body weight are accounted for, this association is no longer present. As the MC4RKO mice used in our study were already obese when bone parameters were measured, it is difficult to assess the role of decreased melanocortin signaling in regulating bone mass independent from increases in body weight. It has been suggested that the elevated bone mass in MC4RKO mice arises from elevated cocaine and amphetamine regulated transcript (CART) levels [9]. Indeed, mice lacking the prohormone convertase carboxypeptidase E are obese with low bone mass and are unable to produce mature α-melanocyte stimulating hormone (the primary MC4R ligand) or CART [20]. This suggests that CART signaling lies downstream of the MC4R in the regulation of bone mass. Additionally, there is some evidence to suggest that melanocortin signaling has direct influences on bone. The MC4R is expressed both on osteoblasts and osteoclasts [21], [22], demonstrating the potential for melanocortin peptides to directly influence bone metabolism. While increased bone mass has been previously described in the MC4RKO mouse, our study is the first to show that bone strength is also increased by MC4R deficiency. Importantly, this demonstrates that in addition to regulating bone mass, melanocortin signaling is also an important determinant of bone quality.

To further evaluate the function of skeletal muscle in MC4RKO mice, we assessed treadmill run time as a measure of endurance. MC4RKO mice were markedly deficient in their exercise capacity. Furthermore, increased body weight does not appear to be responsible for this effect, as there was no relationship between body weight and treadmill run times. We also found that grip strength is slightly increased in MC4RKO mice, suggesting a shift in the balance of muscle fiber types. However, there were no differences in fiber type distribution in MC4RKO mice, demonstrating that an overt change in the balance of oxidative and glycolytic fibers is not responsible for the exercise phenotype of these animals. MC4RKO mice also have a dramatically decreased resting heart rate, consistent with previous findings in MC4R deficient mice and humans [23], [24]. It is possible that an inability to achieve peak exercise-induced cardiac output underlies the exercise phenotype of the MC4RKO mouse. MC4RKO mice have normal heart weights and gross appearance [8]. However, the central melanocortin system is fundamental regulator of autonomic outflow to many tissues, suggesting alterations in cardiac sympathetic nerve activity underlie this phenomenon [17], [25]. Indeed, central administration of melanocortin agonists produces a relative tachycardia [26]. However, there is likely some ligand and neuroanaotomic specificity to this effect as others have found that melanocortin agonists produce bradycardia [27]. Interestingly, we found decreased expression of the β1 adrenergic receptor in the hearts of MC4RKO mice, demonstrating that cardiac in addition to CNS changes may underlie decreased heart rate in MC4RKO mice. While a complete mechanistic description of this phenomenon is beyond the scope of this work, it is clear that global deficiency of the MC4R results in a decrease in resting heart rate. Interestingly, previous studies have only demonstrated significant decreases in heart rate during the night cycle when the animals are active, although a trend toward decreased heart rate was observed during the day [23]. Technical considerations resulting in decreased variability likely account for our ability to detect a significant suppression of heart rate in MC4RKO mice during the day (Measurement in anesthetized mice over a short time period vs. average heart rate over 12 hours in freely moving animals). However, the finding that MC4RKO mice fail to appropriately increase their heart rate during a time of increased activity (night cycle) is consistent with heart rate as a determinant of exercise impairment in MC4RKO mice. Another plausible explanation is that the central melanocortin system is also a regulator of glucose uptake by skeletal muscle. Central administration of melanocortin agonists increases the activity of AMP-activated protein kinase (AMPK), which results in the translocation of the type 4 glucose transporter to the cell membrane [16]. Therefore, impaired glucose uptake in skeletal muscle consequent to decreased AMPK activity also has the potential to underlie the endurance defect in MC4RKO mice.

Signaling via the MC4R controls multiple facets of growth and metabolism. While numerous studies have focused on the regulation of fat mass by the central melanocortin system, the regulation of lean mass has not been previously explored. We demonstrate increased lean mass in the setting of MC4R deficiency with an increased bone mass significantly contributing to this finding. Furthermore, MC4R deficiency results in impaired exercise performance. This result has important clinical ramifications for the care of patients with the melanocortin obesity syndrome, where reduced ability to sustain physical activity may confound attempts to control body weight via exercise.

## Materials and Methods

### Ethics Statement

Experiments were conducted in accordance with the National Institutes of Health Guide for the Care and Use of Laboratory Animals, and approved by the Animal Care and Use Committee of Oregon Health & Science University, Protocol Number A931.

### Animals

Wild type C57BL/6J mice (20–25 g) were obtained from The Jackson Laboratory (Bar Harbor, ME). MC4RKO mice were used as previously reported [2]. MC4RKO mice were backcrossed at least 10 generations into the C57BL/6J strain. All animals were maintained on a normal 12∶12 hr light/dark cycle and provided *ad libitum* access to water and food (Purina rodent diet 5001; Purina Mills, St. Louis, MO). The high fat diet (HFD) (D12492, Research Diets, Inc.) contains 60% fat (5.24 kcal/g). Mice were sacrificed by decapitation under anesthesia from a ketamine cocktail.

### Body composition

Mouse body composition analysis was performed using a 4-in-1 small animal MRI (Echo Medical Systems, Houston, TX).

### Treadmill

Exercise performance was measured with an Exer-3/6 treadmill (Columbus Instruments, Columbus, OH). Animals were conditioned in the treadmill chamber every other day for one week prior to the experiment. At the start of training sessions, animals were acclimatized to the treadmill chamber for ten minutes with the treadmill off. The speed of the treadmill was started at 6 m/min was increased by 1 m/min every min. Once 10 m/min was reached, the animals were run for 5 minutes. On the day of the experiment, the treadmill was started at 6 m/min, and the speed was increased by 1 m/min every 10 minutes until 15 m/min was reached. Animals were run at 15 m/min until exhaustion.

### Real Time PCR

Heart RNA was extracted using the RNeasy fibrous tissue mini kit (Qiagen, Valencia, CA) according to the manufacturer's instructions. cDNA was transcribed using Taqman reverse transcription reagents and random hexamers according to the manufacturers instructions. PCR reactions were run on an ABI 7300, using Taqman universal PCR master mix, using Taqman gene expression assays: Adrb2 (Mm02524224_s1), Adrb1 (Mm00431701_s1) Chrm2 (Mm01701855_s1), Adrbk1 (Mm00804778_m1) (Applied Biosystems, Carlsbad, CA). Relative expression was calculated using the delta-delta Ct method, and was normalized to vehicle treated control. Statistical analysis was performed on the normally distributed delta-Ct values.

### Fiber type analysis

For analysis of muscle fiber type, 9 µm unfixed cryosections of gastrocnemius were blocked for 1 hour in PBS/1% BSA/10% goat serum, and then incubated overnight in primary antibody diluted 1∶250 in PBS/1% BSA/10% goat serum. The following primary antibodies were used: SC-71 for myosin IIa, BF-F3 for myosin IIb (Developmental Studies Hybridoma Bank, University of Iowa, Iowa City, IA), and anti Myosin I (Vector Labs, Burlingame, CA). Sections were washed in PBS/0.025% triton-X-100, and incubated with a goat anti-mouse Alexafluor-488 nm labeled secondary antibody (Invitrogen, Carlsbad, CA) diluted 1∶500 in PBS/10% BSA. Sections were mounted with Vectashield fluorescent mounting media (Vector Labs, Burlingame, CA). Images were acquired on a Leica DM4000 B microscope, using a Leica DFC340 FX camera at ambient temperature at 100× magnification using Leica Applications Suite 3.6 software (Leica, Buffalo Grove, IL).

### Grip Strength

Grip strength was measured using a Grip Strength Meter (Columbus Instruments, Columbus, OH). Five measurements were collected per animal. The high and low value for each animal were discarded, and the middle three values averaged.

### Heart rate

Mouse heart rate was measured using a Vivo770 (Visualsonics, Toronto, ON) under isoflurane anesthesia. Mice were maintained under anesthesia for one hour prior to heart rate measurement to establish a stable baseline.

### Bone Phenotype

Female mice from the wild type (WT, C57BL/6) and MCR4KO strains were bred under identical conditions. All procedures were approved by the VA Institutional Animal Care and Use Committee and performed in accordance with National Institutes of Health guidelines for the care and use of animals in research.

At 5 and 10 months of age, both WT and MCR4KO mice were euthanized by CO_2_ inhalation and weighed to the nearest 0.1 g. Lumbar vertebrae and femora were immediately harvested, wrapped in sterile gauze soaked in PBS, and stored frozen at −20°C for subsequent biomechanical analyses. Prior to sacrifice, *in vivo* whole body composition and BMD measurements were carried out on anesthetized mice using dual-energy X-ray absorptiometry (pixiMus®, GE Medical Systems, Waukesha, WI) after an overnight fast. Analysis was performed using the mouse whole body software provided by the manufacturer. BMD of isolated femoral and L_5_ vertebral specimens were determined as well.

Cortical femoral shaft bone geometry was assessed with a desktop x-ray microtomographic scanner (SkyScan Model 1074, Aartselaar, Belgium). Images were analyzed with Optimas software (version 6.2; Media Cybernetics, Silver Spring, MD). To determine femoral structural properties, the left femur was tested to failure by three-point bending on a high-resolution materials test apparatus (Model 4442, Instron Corp., Canton, MA). Load and displacement data were recorded and failure load was determined using system software. Vertebrae were evaluated using a desktop *μ*CT imaging system (*μ*CT40; Scanco Medical AG, Bassersdorf, Switzerland) equipped with a 10-mm focal spot microfocus X-ray tube and images acquired with a 12 µm isotropic voxel size, as previously described [28]. Morphometric variables describing bone microstructure were computed using direct 3D methods, including bone volume fraction (BV/TV, %), trabecular number (Tb.N, mm^−1^), trabecular thickness (Tb.Th, µm), trabecular separation (Tb.Sp, µm), connectivity density (ConnD, mm^−3^), and the structure model index (SMI), a measure of the plate- versus-rod-like nature of the trabecular structure. All microCT analyses adhere to published guidelines [29].

### Statistics

Data are expressed as mean ± SEM. Statistical analysis was performed using Prism software (Version 4.0, Prism Software Corp., Irvine, CA). All data were analyzed with an unpaired t test, or Pearson correlation. For all analyses, significance was assigned at the level of P<0.05.
